# A synopsis of the Joint Environment and Human Health Programme in the UK

**DOI:** 10.1186/1476-069X-8-S1-S1

**Published:** 2009-12-21

**Authors:** Michael N Moore, Pamela D Kempton

**Affiliations:** 1 Plymouth Marine Laboratory, Prospect Place, the Hoe, Plymouth, PL1 3DH, UK; 2 Natural Environment Research Council, Polaris House, North Star Avenue, Swindon, SN2 1EU, UK

## Abstract

The Joint Environment and Human Health (E&HH) Programme has explored how both man-made and natural changes to the environment can influence human health. Scientists have tackled the complicated mix of environmental, social and economic factors that influence health, particularly focusing on naturally occurring toxins, man-made pollutants, nanoparticles and pathogens to see:

• how they spread within the environment

• how their properties change as they interact with other substances or organisms

• how we become exposed to them, and

• their impact on human health.

The Programme has not only succeeded in bringing together scientists from a broad range of environmental, social and biomedical backgrounds, but also fostered new relationships with end users and policy makers. This new community is helping to provide the multidisciplinary capacity able to respond in an interdisciplinary way to resolve problems that are intrinsically interfacial in character. Many of these questions relate to complex issues such as the environmental biology and geochemistry of soils and how these influence the transport, accessibility and bioavailability of chemical pollutants and infectivity of pathogens. The dispersion of harmful particles in the atmosphere is another area of major concern where the E&HH Programme has broken new ground by showing how the chemical and physical properties of such particles influence their environmental behaviour and may govern their toxicity and resultant pathological reactions induced following inhalation. Working groups and networks have identified potential health problems concerning the transport and emergence of human pathogens associated with food, soil, air and water. The consequence(s) of global and regional climate change for the environmental behaviours of pollutants and pathogens have been considered by a number of the projects supported by the E&HH programme.

The selection of articles in this supplement reflect the broad scope of the E&HH programme. By effectively identifying and interconnecting these interdisciplinary elements, the E&HH programme has fostered the emergence of new ways of solving problems in areas of research that have, until recently, had little connection with one another. This has not only helped build new research groupings, but has also led to exciting new scientific developments as described in this issue of *Environmental Health.*

## Introduction

The natural environment contributes significantly to people's health through the quality of air we breathe, the food we eat and the water we drink. On the one hand, it offers health enhancing economic and recreational opportunities, while on the other, it is threatened by activities such as transport, industrial processes, agricultural and waste management practices. Indeed, humans have altered, and will continue to alter, their environment, while remaining dependant upon ecosystems as resources of food, water and materials (e.g., timber, biofuels etc.). Evaluation and management of the resultant impacts, on both ecosystems and human health, have largely been undertaken as separate activities, under the auspices of different disciplines with no obvious interaction. Hence, many of our perceptions of the relationships between the natural environment and human health are limited and still relatively unexplored, leaving critical knowledge gaps for those seeking to develop effective policies for sustainable use of resources and environmental and human health protection [1].

If society is to have a practical capability to forecast how environmental change impacts on linkages between natural systems, social systems and human health, we must develop a better understanding of the functioning of the biosphere and our connections with it [1]. The difficulty is that living systems are strongly interdependent, often in ways that continue to surprise us, and environmental impacts -- by both natural events and man-made interventions -- are a fact of life. Among these impacts are harmful environmental pollutants and potentially pathogenic organisms to which people are exposed through a variety of complex transport and exposure pathways, e.g. through air, soil, water and from chemical and microbiological residues in food. How exposed populations respond to these stressors in both the short and long term will depend on both the degree of exposure and on individual factors such as socio-economic and nutritional status, age, genes, gender and behavioural aspects that influence avoidance or risk-accepting attitudes [1].

Developing the capacity to predict and minimise these impacts -- and their harmful consequences for biological resources, ecosystems and human health -- is a daunting task for environmental (and other) legislators and regulators. Complicated issues are involved in evaluating environmental risk, such as the effects of the physico-chemical interactions on the speciation and uptake of pollutant chemicals, inherent inter-individual and inter-species differences in vulnerability to toxicity, the emergent toxicity of complex chemical mixtures, and knowledge of reservoirs of microbial pathogens as well as factors governing infectious viability. It is, therefore, not surprising that these are areas of growing public and government interest, which are reflected in UK and international priorities: for example in the Environment Research Funders' Forum, the EU, the World Health Organisation (*Millennium Ecosystem Assessment*, 2005), and International Year of Planet Earth 2006 (Earth & Health theme).

While such predictive capability must remain a major longer-term scientific goal, the scope of the Environment and Human Health programme in its first phase has been of necessity more pragmatic, with a focus on exploratory ("proof-of-concept") research and building the interdisciplinary community required for the task. A particular emphasis in the programme has been in trying to bring the research community together with policy makers and decision makers in order to foster the process of policy formulation through improved knowledge transfer and exchange.

This supplement in *Environmental Health *brings together some of the first outputs from the E&HH programme. The papers presented here clearly demonstrate the breadth of the programme and underline the successful interfacial investigations that have been undertaken. User organisations have been involved in many of these projects, either as co-investigators or as active participants at workshops and mini-conferences. The success of the interdisciplinary approach has resulted in exciting new findings, and also raised many novel research questions, that would probably not have emanated from the more traditional single discipline or limited multidisciplinary approach.

## The Environment and Human Health Programme

In recognition of the research challenges described above, the Natural Environment Research Council undertook a series of consultation events involving both stakeholders and the academic community. One outcome of these consultations was the identification of four broad areas of research that were considered to provide the focal points for the most effective way forward in addressing the very complicated environmental, health and social issues:-

1. *pathogens*

2. *pollutants *(particles and chemicals)

3. the *pathways *these follow through the environment

4. and their interactions with *people*

The joint Environment and Human Health (E&HH) programme was formulated to address these areas, in the context of the overarching question of **'How do we effectively manage the natural environment to improve human health?'**

A multidisciplinary research approach would clearly be needed, which in turn required collaboration across a range of funding organisations. E&HH ultimately brought together nine different funders, including UK Research Councils (the Natural Environment Research Council, Economic and Social Research Council, Medical Research Council, Biotechnology and Biological Sciences Research Council and the Engineering and Physical Sciences Research Council), Government Departments (DEFRA, the Environment Agency, the Ministry of Defence) and a charity (Wellcome Trust).

**Figure 1:**
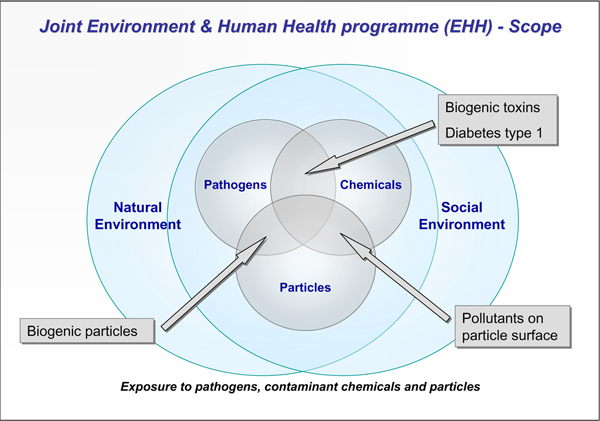
**Diagram showing the scope of the Environment & Human Health Programme, its interfacial nature of the research and the focus on transport and effects of pollutant chemicals, particles and pathogens**. Future research challenges are indicated in the interfacial areas between particles, chemicals and pathogens, which include biogenic toxins from cyanobacteria and algae, harmful particle coatings of microbial origin, and indications that the complex aetiology of type 1 diabetes may involve both viral infection and environmental chemical exposure [14,16,17.]

In its first phase, the principal objectives of the Environment and Human Health programme have been to (i) identify and prioritise the research needs within the four broad areas identified above and (ii) encourage, grow, develop and facilitate the research community required to tackle the "real-world", inter and multi-disciplinary problems faced not only in the UK but globally. Therefore, E&HH has aimed to:

- create a constituency for both inter- and multi-disciplinary work that would underpin our understanding of the links between the environment and human health

- provide better knowledge that would improve our ability to identify and predict emerging issues of potential concern (Figure 1)

- improve the evidence base for risk assessment and regulation of activities needed by government departments and agencies, and other stakeholders

with the aim of improving the health of people, both in the UK and globally.

### Themes & approaches

Identifying and prioritising the research needs was a principle objective for E&HH in its first phase. Using information collected during the various consultations, the E&HH Science Advisory Committee identified a number of potential areas of interest, and these are summarised below.

Transport and dynamics of micro-organisms of human health importance in the natural environment

• response of microbial transfers to environmental change

• science based issues of scaling up from the local site to the catchment or appropriate policy level

• gene flow (e.g. involving antibiotic resistance) through the microbial horizontal gene pool

• harnessing a systems biology approach to help understand complex processes in the soil microbial environment where indirect impacts on human health may result in terms of surface water quality or bathing water quality

• interactions of microorganisms and pollutants

• integration and quantification of risks to humans through both environmental and other pathways

• the risks of organic agriculture (including trans-national transport of pathogens) through different approaches to crop and animal production

Emerging infectious diseases

• risk assessment, the use of indicators, and anticipatory modelling of novel pathogen dynamics

• influence of global and local environmental change (e.g. climate change, nitrogen deposition, deforestation; as well as land use change, for example CAP reform and the EU Water Framework Directive)

• ecology of wildlife reservoirs and vectors in emergent diseases

• role of farm workers in disease transmission

Transport and dynamics of both chemicals and particles of different sizes and compositions in the natural environment that are of human health importance

• assessment of exposure and bioavailability from various physical (soil, water, air, food) and behavioural pathways through different routes (e.g. developing and using effective biomarkers) to better inform toxicology, epidemiology and human risk assessment

• active features of particles that cause problems, e.g. surface properties, size and composition

• interactive effects of mixtures of chemicals in the environment and the impact on human health, exploiting sensitive analytical and molecular techniques

• chronic low level exposures to toxins, leading to human health effects including trans-generational toxicity (genetic and epigenetic) and other long term outcomes

• inter-individual susceptibility to environmental factors and interactions (e.g. toxicity), including genetic make up, particularly with respect to susceptible groups such as foetus, children, elderly, and those with ill health or receiving medication; including the extent to which these differences may be socially structured

• effect of changes in the environment (e.g. climate change, land use change) on human health; regulatory changes leading to land use change and impacts of changes on the pollution profile and nutrient depletion

• soil degradation and trace metal deficiencies affecting human health

Technologies providing new capabilities for establishing and predicting the impact of the environment on human health

• application of new techniques including computational, physical, engineering, analytical chemistry/biochemistry methods, i.e. application of massively parallel screening approaches possibly using new lab-on-a-chip methods for understanding the interplay of pathogens/toxic agents with other environmental agents on human health.

• novel techniques for studying pathogenic microbes or pollutants (chemicals or particles) in the environment where a potential link to human health may be important.

• approaches to enable an understanding of the impact of nanotechnology and nanoparticles on human health.

• data analysis/modelling tools, including approaches such as machine learning or other intelligent agents, possibly providing predictive capability from large data sets gathered from social, environmental or medical studies. It is anticipated that these tools could provide predictive models of relevance to human health, or alternatively provide models for fate and transport.

Social, economic and behavioural factors in the genesis and health impact of environmental hazards

• what are the macro-social factors and processes for example, business organisation, trade, urbanisation and population change, influencing the exposure of people to environmental risks and hazards (pathogens and pollutants)?

• what is the role of factors such as socio-economic status, age, gender, and culture in shaping behaviours relevant to environmental health risks?

• how does a stressful social or physical environment impact on biological processes linking the environment and disease?

• what is the importance of age, culture, social position, disability, and illness for resilience and adaptive capacity in the face of environmental health hazards?

• what is the impact of differing perceptions of risk and attitudes in enhancing public engagement and dialogue about environment and health issues?

• how are political, economic, cultural and social forces shaping the emergence of new environmental health risks and benefits and how may these be managed?

• can we quantify the benefits to human health of changes in the environment such as the spatial distribution of and access to green space?

• what are the economic and social costs (or benefits) of environmental impacts on health?

### Building a new community

In order to successfully tackle the research priorities identified above, collaborations among scientists that had traditionally not worked closely together needed to be facilitated. Consequently, the focus of the E&HH Programme has been on **capacity building **and supporting inter-disciplinary activities such as:

• proof of concept studies or exploratory awards

• workshops/networks

• working groups

• "discipline hopping" opportunities to encourage career development for young scientists.

The £4.4 m E&HH programme currently supports 37 projects that cover a broad range of environmental concerns linked to human health. They address a complicated mix of environmental, social and economic factors that influence health, particularly focusing on naturally occurring toxins, man-made pollutants, nanoparticles and pathogens to ascertain:

• how they spread within the environment

• how their properties change as they interact with other substances or organisms

• how humans become exposed to them, and

• their impact on human health.

There are also projects investigating the effects of micronutrient deficiencies in agricultural soil on maternal and post-natal health in Malawi, the potential beneficial effects of an association with nature and human well-being (biophilia), and plant virus infection as a determinant of pollen allerginicity.

The perturbation of environmental interactions as a consequence of global climate change is an important sub-theme running through many of the E&HH supported projects on pollutants, particles and pathogens.

Collectively, these projects have brought together scientists from many disciplines including environmental and social science, medical, biomedical and public health research. They have also interacted closely with government departments, agencies and industry. Sharing such diverse knowledge and skills has enabled a holistic view on how the environment influences human health.

Some specific achievements arising from the interfacial research that characterise the E&HH programme are presented below, and these underline why the problems being addressed by this programme, can only be addressed by multi- and interdisciplinary groups of scientists working together in cohesive collaborative projects. -

1. Silver nanoparticles are known for their antibacterial properties (exploited in wound dressings), but the impact of their release into the environment has been largely unknown. The work of Gaiser et al. demonstrates the toxicity of silver nanoparticles in a human food-chain model including invertebrates, fish and mammals [2].

2. Inhalation of endotoxin at elevated concentrations has been associated with acute airway obstruction, hypersensitivity pneumonitis, chronic bronchitis and decreased lung function. Although waste management activities are acknowledged as a source of environmental endotoxins, causing occupational exposure, little information regarding endotoxin dispersal from green waste composting, and the potential for non-occupational exposure, has been available. Deacon et al. demonstrate that bacterial endotoxin associated with airborne particles produced by commercial composting have detrimental biological effects on an *in vitro *cell-based model of the lung [3]

3. Collins et al. have developed a robust analytical system for the assessment of the bioaccessibility of arsenic and polycyclic aromatic hydrocarbons (PAHs) has been developed in a simulated human gut environment, resulting in the development of a patentable test kit [4].

4. Global food insecurity is associated with micronutrient deficiencies and it has been suggested that 4.5 billion people world wide are affected by deficiencies in iron, vitamin A, iodine and zinc. The most vulnerable are young children and women of childbearing age. A pilot study is underway in Southern Malawi that attempts to link the geochemical and agricultural basis of micronutrient supply through spatial variability to maternal health and associated cultural and social aspects of nutrition [5].

5. Information on environmental contamination with viruses, or their potential for persistence in the environment or in the food chain, is currently incomplete. A network funded by the EHH programme has improved our understanding of the environmental pathways for exposure to pathogenic viruses and emerging viral problems (e.g., from farm animals and global transport of food products from industrial organic farms in China, Mexico and California). A report by Cook et al. entitled "Current Knowledge Gaps Regarding Transmission of Viruses through the Environment and Food in the United Kingdom" has been submitted to NERC; reference NE/E009026/1.

6. MRSA and other drug-resistant microbes are no longer just a problem in the hospital environment. They are present in the natural environment, with the attendant concerns about the transfer of the genes conferring drug-resistance to other species of bacteria [6]. Research supported through the EHH programme has led to development of screening tests for MRSA in the agricultural environment.

7. Lacharme-Lora et al. show that human disease causing bacteria can survive inside free-living helminth worms (nematodes) and are protected from the action of chemical sanitiser treatment [7]. Food-borne diseases are a significant public health problem where pathogens such as *Salmonella *occurring in the soil can interact with such organisms. The inference is that soil nematodes can provide a protective micro-environment for human pathogens with health implications for the persistence of such pathogens in the soil.

8. McNally et al. show evidence of space-time clustering amongst females for cases of type 1 diabetes diagnosed in north-east England, and clustering was confined to cases from more densely populated areas. These findings are consistent with a possible aetiological involvement of an infectious agent [8].

9. Research by Givens et al. and others [9,10] suggests that the health benefits of organic foods may be over-rated, although further investigation is essential before making critical comparisons with normal foods. This project dealt with a complicated set of potentially interacting factors including soil science, pesticide toxicology, biogenic fungal toxins and infectious pathogens, as well as the nutritional composition of the foods in question.

10. Climate change is likely to increase human exposures to agricultural contaminants. The magnitude of the increases will be highly dependent on the contaminant type. Risks of many pathogens, particulate and particle-associated contaminants could increase significantly. However, Boxall et al. suggest that these increases in exposure can, for the most part, be managed through targeted research and policy changes [11].

11. Richardson et al. are working to derive a summary measure of multiple environmental deprivation at a small area level, akin to the measures of multiple socioeconomic deprivation used by health researchers from many disciplinary backgrounds [12]. The key achievements have been to (i) systematically identify which elements of the physical environment should be included in the measure, (ii) compile appropriate environmental data for the whole UK, (iii) produce several different versions of the measure (iv) discover independent associations between the measures and variation in all cause and cause specific mortality, and (v) begin to examine the interaction between social and physical environments on health inequalities in the UK.

## Concluding remarks

While the health of the UK, and other parts of the world, has improved considerably over the last three decades, there remains considerable social and spatial difference in ill health distribution. Causal factors have been identified that explain much of this difference for certain diseases (e.g. high cholesterol and insufficient exercise for coronary heart disease). However, a significant proportion of this difference in health burden remains unexplained, such as in cardio-vascular disease, diabetes, obesity and many cancers where environmental factors are likely to be significant [13-19]. Furthermore, we are only beginning to appreciate the possible impact of changes in the climate and global environment on ecosystems and health.

Undoubtedly, new developments and improvements in our scientific understanding of how environmental change impacts on the linkages between ecological integrity, environmental goods and human health will aid us as we seek to develop an acceptable standard of living for many more people. This will in turn help us to ensure that the ecological pillars, which support our society and industries, are protected and remain sustainable. We must also aim to successfully integrate social and natural systems on a local scale, while understanding the larger scale ramifications and consequences of decisions on national and trans-national scales.

The Joint Environment & Human Health Programme has played a significant role in taking this agenda forward, not only by bringing together researchers from a broad range of environmental, social and biomedical backgrounds, but also by fostering new relationships with end users and policy makers. This new community is helping to provide the multidisciplinary capacity able to respond in an interdisciplinary way to resolve problems that are intrinsically interfacial in character (Fig. 1).

Many of these issues relate to complex problems such as the environmental biology and geochemistry of soils and how these influence the transport, accessibility and bioavailability of chemical pollutants and infectivity of pathogens. The dispersion of harmful particles in the atmosphere is another area of major concern where the E&HH Programme has broken new ground by showing how the chemical and physical properties of such particles influence their environmental behaviour and may govern their toxicity and resultant pathological reactions induced following inhalation.

**Figure 2:**
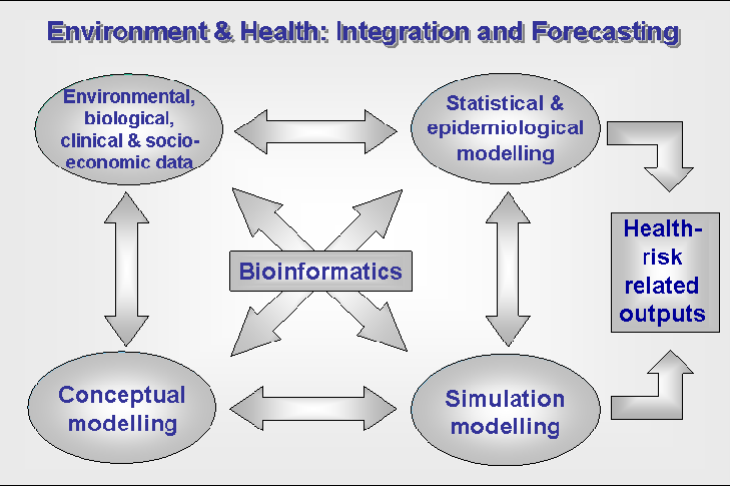
**Holistic systems approach to studying environment and health problems involving multi- and inter-disciplinary biomedical and environmental research**. This process-based synthesis facilitates the integration of environmental, biological, ecological, epidemiological and socio-economic data for forecasting risk. To be effectively interdisciplinary the multidisciplinary groupings in a research project need to be highly interactive in addressing the scientific problem.

A significant aspect of the E&HH Programme has been inclusion of broader socio-economic issues involving people-orientated environmental health-related problems (Figure 1 &2). Unfortunately, there remains a relative dearth of substantial epidemiological data that would permit a comprehensive understanding of possible causal links between human and ecosystem health (see - *Millennium Ecosystem Assessment*, 2005, http://www.millenniumassessment.org/en/index.aspx; and World Health Organization, http://www.who.int/topics/environmental_health/en/). However, by effectively identifying and interconnecting the interdisciplinary elements, we are beginning to see the emergence of new ways of solving problems in what are, at present, areas of research that have traditionally had little connection with one another (Figure 2). The E&HH Programme has clearly demonstrated the value of supporting the exciting and novel integrative and holistic research which has resulted from the interdisciplinary research groups that it supported (Figure 2). The success of the programme has fostered the evolution of two new successor programmes (i.e., *Environmental and Social Ecology of Human Infectious Diseases- *ESEI; and *Environmental Exposure and Health*- EEHI).

## Successor programmes to environment and human health

The UK Natural Environment Research Council (NERC), Medical Research Council (MRC), Economic and Social Research Council (ESRC) and Biotechnology and Biological Sciences Research Council (BBSRC) are supporting two new programmes of research that will tackle the major roles environmental and social factors play in human health.

The vision for these initiatives is the establishment of truly interdisciplinary teams of researchers, conducting high quality state-of-the-art innovative research, addressing national/international research priorities that will inform and impact on policy and practice. The themes for these programmes are outlined briefly below.

### Environmental and social ecology of human infectious diseases

The emergence (and re-emergence) of virulent pathogens remains an ongoing threat to human health. This new initiative aims to establish novel inter-disciplinary approaches to studying the ecology of infectious diseases, in order to better anticipate, prepare for, and control future outbreaks. A holistic systems approach will be required, which takes into account the ways in which the natural and social environments affect the emergence (emergence, re-emergence, and development of drug resistance) and spread of infectious disease. Since most emerging infections are zoonotic, there will be a particular focus on the animal reservoir as a source of infectious disease and how animal pathogens spill over into human populations and spread through communities in the UK and other parts of the world.

### Environmental exposure and health

Vital ecosystem services that sustain life and health are increasingly under pressure from population growth and urbanisation. Adverse health effects result from the degradation of these services in part because water, land, food or air are more often contaminated with pollutants, such as endocrine disruptors, pesticides, drugs, and particles. Furthermore, these stressors often occur in combination with other environmental and dietary stressors, such as increased temperature and a diet low in anti-oxidants. The aim of this new initiative on Environmental Exposure and Health is to provide important new knowledge on the interconnections and pathways between environmental pollutants and stressors, exposures, early effects (for example, biomarkers) and health outcomes in humans, including variations in susceptibility and the definition of health risks. This integrated understanding is vital to inform development of evidence-based policies.

## Competing interests

The authors declare that they have no competing interests.

## Authors' contributions

Mike Moore is the Science Co-ordinator for the Joint E&HH and ESEI Programme; and Pamela Kempton is the Programme Manager and Head of Research for the UK Natural Environment Research Council. Both authors read and approved the manuscript.
